# 229. mRNA-1010, an mRNA-Based Influenza Vaccine, is Safe and Efficacious in Adults Aged ≥50 Years

**DOI:** 10.1093/ofid/ofaf695.012

**Published:** 2026-01-11

**Authors:** Elissa Malkin, Anita Kohli, Rebecca Clark, Isabel Leroux-Roels, Evelyn Du, Agi Buchanan, Bryony Hicks, Eleanor Wilson

**Affiliations:** Moderna Therapeutics, Inc., Cambridge, Massachusetts; The Institute for Liver Health Arizona, Tucson, Arizona; Layton Medical Centre, Blackpool, England, United Kingdom; Ghent University and Ghent University Hospital, Ghent, Belgium, Ghent, Oost-Vlaanderen, Belgium; Moderna, Inc, Cambridge, Massachusetts; Moderna, Inc., Cambridge, Massachusetts; Moderna, Inc, Cambridge, Massachusetts; Moderna, Inc., Cambridge, Massachusetts

## Abstract

**Background:**

mRNA-1010, a novel mRNA-based influenza vaccine targeting vaccine-matched influenza A and B strains, has demonstrated superior immunogenicity compared to licensed standard-dose (SD) and high-dose comparators, as measured by hemagglutination inhibition assay.^1^ We present the safety and relative vaccine efficacy (rVE) from the end of influenza season analysis of the pivotal phase 3 trial comparing mRNA-1010 to SD influenza vaccination in adults ≥50 years.

**Figure.**
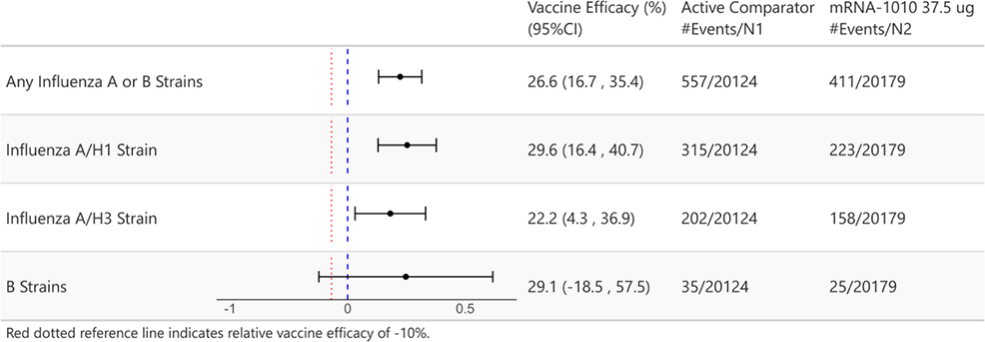
Forest Plot of rVE Against RT-PCR–Confirmed Protocol-Defined ILI by Any Influenza A or B Strain and by Vaccine-Contained Strains rVE is defined as 100 × (1 - hazard ratio [mRNA-1010 vs Active Comparator]). The rVE and the CI are based on a stratified Cox proportional hazards model, with the study vaccination group as a fixed effect, adjusting for the randomization stratification factors (age [50-64, 65+ years] and previous influenza season vaccination status).

**Methods:**

This phase 3, randomized, double-blind, active-controlled study (NCT06602024) enrolled participants (≥50 years) from 11 countries throughout the Northern Hemisphere. Participants were randomized 1:1 to receive a single dose of trivalent mRNA-1010 37.5 µg (12.5 µg hemagglutinin mRNA per strain) or SD comparator (Fluarix 45 µg or Fluarix Tetra, Influsplit Tetra, or Alpharix Tetra 60 µg). The primary efficacy endpoint was rVE in prevention of the first episode of RT-PCR–confirmed protocol-defined influenza-like illness (ILI) caused by influenza A or B strains beginning ≥14 days after study vaccination through end of the influenza season.

**Results:**

In the 2024-2025 influenza season, 40,703 participants were randomized and received study vaccine (mRNA-1010, n=20,350; SD comparator, n=20,353). The median age was 64 years (range 50-97 years); 56.9% were female; 82.6% were White, 13.2% Black; and 10.4% Hispanic/Latino. Median follow-up was 181 (1-227) days. Solicited adverse reactions within 7 days of vaccination were more frequently reported in the mRNA-1010 than the SD comparator group; most reactions were mild/moderate, transient, and self-limiting. mRNA-1010 demonstrated an rVE of 26.6% (95% CI, 16.7-35.4%) against RT-PCR–confirmed protocol-defined ILI compared to SD comparator, meeting the prespecified superiority criteria (lower bound of the 95% CI >9.1%; 1-sided p=0.0005; Figure).

**Conclusion:**

mRNA-1010 demonstrated superiority over SD comparator in the prevention of RT-PCR–confirmed protocol-defined influenza disease in adults ≥50 years, consistent with approved enhanced influenza vaccines. rVE was consistent across vaccine-included strains, including Influenza B strains.

References:

1. Soens M, et al. Vaccine. doi:10.1016/j.vaccine.2025.126847

**Disclosures:**

All Authors: No reported disclosures

